# Supplementary material for: Are carbon emissions trading and green financial instruments synergistic? -Comprehensive quantitative research based on content analysis
Source: PLoS One. 2024 Mar 7;19(3):e0298601. doi: 10.1371/journal.pone.0298601 (PMC10919601; doi:10.1371/journal.pone.0298601)
Supplement: S1 Data — (ZIP) [file pone.0298601.s001.zip › Data/地级市Z_F工作报告.zip!/╜¡╬≈/╕╙╓▌╩╨/└┤╫╘╣1⁄2╓┌║┼í╛┬φ┐╦╩2╛▌═°í┐_2009.docx]

各位代表：

　　我代表市人民政府向大会作政府工作报告，请予审议，并请各位政协委员和其他列席人员提出意见。

　　一、2008年经济社会发展回顾

　　2008年，在中共赣州市委的正确领导下，在市人大、市政协的监督和支持下，市政府突出践行科学发展观，紧紧依靠全市人民，纵深推进“对接长珠闽，建设新赣州”发展战略，抓好“一条主线，六个重点”工作，较好地完成了市三届人大二次会议确定的全年目标任务。预计生产总值突破800亿元，达到831亿元，按可比价格计算增长13.0％。财政总收入突破100亿元，达到100.13亿元，地方一般预算收入55.47亿元，分别增长34.4%和42.5%。财政总收入占GDP的比重达到12%，税收占财政总收入的比重达到85.2%。

　　十大体系建设成效显著，各项改革不断深化。十大体系政策框架日趋完善,新出台推进承接产业转移等政策文件27个。新建成40个物质载体项目，43个在建物质载体项目进展顺利。十大体系进园区、进市场、进社区富有成效，服务园区企业扩招员工7万人，新增贷款32.8亿元，进出口企业物流成本降低30%。电子政务统一应用平台和政务信息网扩容工程全面启动。我市制定的《脐橙》国家标准正式实施。新组建地方铁路公司、水务集团和现代服务业公司，对矿产资源管理机构实行市管县垂直管理。一批存量国有企业改革全面启动，市国资委出资监管的企业资产总额增至210亿元，行政事业单位经营性资产收益增长69%。国库集中支付制度全面推行，所有县（市、区）和乡镇实行“乡财县管”。赣州投资环境明显改善，获“粤商最佳投资城市”、“浙商最佳投资城市”和“深港企业最佳投资城市”称号。非公有制经济活力增强，创造税收占财政总收入比重达到58%。

　　新农村建设全面推进，农业农村经济稳步发展。新增新农村建设点2451个，累计完成改水8.88万户，改厕8万座，建设通村组水泥公路1975.8公里。圩镇环境整治取得新进展。实现农业总产值281亿元，增长4.7%；粮食总产量268.5万吨，增长2%。脐橙面积153万亩，产量突破100万吨，加工甜橙开发整地面积10.2万亩。生猪、蔬菜、花卉苗木、烟叶稳步发展。新增国家、省级龙头企业2个和5个。年销售收入500万元以上的农业龙头企业248个。新增登记注册农民专业合作社309个。251座二期规划内病险水库除险加固基本完成，54座中央专项规划内病险水库除险加固已开工。农业机械化综合水平提高4.5个百分点。组织530名高校毕业生到农村“三支一扶”，积极开展“百名专家为新农村建设服务”活动。培训农民50.2万人次。成功举办中国（赣州）第六届脐橙节。

　　重大项目建设势头强劲，基础设施日臻完善。预计完成全社会固定资产投资400亿元，增长33%。60个重点工程项目在建55个，完成投资140亿元，实现增长翻番，开工项目数和投资额均创历史新高。新黄金机场、赣州出口加工区、华能瑞金电厂一期等重大项目竣工。赣州南500KV输变电工程和厦蓉高速瑞赣段、济广高速鹰瑞段、泉南高速石吉段、赣大高速茅店至三益段等四条高速公路顺利开工。赣龙铁路复线、昌吉赣城际铁路、鹰瑞汕铁路、赣井铁路等项目前期工作加快推进。完成国省道、市养公路及战备公路改造236.8公里，建成农村公路3308.2公里，全市行政村通公路率95.7%，通水泥路率75.5%。启动渡改桥项目67个。17个县（市）污水处理设施全部开工建设。

　　城镇化进程加快，特大城市构建开局良好。全市城市化率达37.3%，比上年提高2.1个百分点。新增城市建成区面积13.5平方公里，县城以上城市新增人口16万人。中心城区构建特大城市全面启动，《赣州市城镇体系规划》、《土地利用总体规划》修编工作进展顺利，六大片区分区规划编制完成。中心城区安排城建项目204个，青少年活动中心、游泳馆、“123”工程、杨梅渡公园一期、章江北岸截污干管工程和章江沿岸夜景一、二期工程等35个项目竣工或基本竣工，章江大桥、飞龙岛大桥、赣江大桥分别完成总投资三分之一以上，中央生态公园、飞翔路整体改造工程、章江新区农民返迁房建设加快，兴国路综合公交枢纽中心投入使用。赣州被国家住房和城乡建设部正式命名为“国家园林城市”。廉租房建设、分配、管理经验被誉为“赣州模式”在全国推广。中心城区公共交通秩序整治工作取得阶段性成效。

　　产业集群进一步壮大，工业经济持续快速增长。规模以上工业实现增加值230亿元，利税总额75亿元，分别增长23.5％和20％；工业增加值占GDP比重36.3%，比上年提高2.2个百分点。新增规模以上工业企业124家，主营业务收入过亿元企业净增50户，其中过10亿元企业净增3户。钨、稀土、萤石新一轮资源整合全面展开，三大优势产业集群实现主营业务收入260亿元，增长26％。全市投资10亿元以上在建工业项目10个，新增6个。投资20亿元的五矿稀土正式开业，金力永磁10亿元风力发电项目将于今年5月投产，中国华星50亿元赣州氟化工基地、广晟12亿元龙南稀土发光材料和光源器具等项目签约。园区规模以上工业实现增加值170亿元，增长30%；利税总额50亿元，增长45%。

　　金融、旅游、物流业加快发展，现代服务业活力增强。第三产业实现增加值302亿元，增长11%。社会消费品零售总额266亿元，增长23%。金融机构存款和贷款余额分别达到882.19亿元和425.35亿元，信贷增量居全省前列。赣州银行县级分支机构扩充至6家，南康赣商村镇银行运作良好。双胞胎集团、章源钨业等企业上市工作取得进展。接待国内外游客人数增长18.9%，旅游总收入增长18.4%。举办赣粤闽千里客家文化旅游长廊系列活动，瑞金叶坪红色旅游景区、赣县客家文化城、定南九曲度假村成功创建4A旅游景区。一批物流项目抓紧建设。航空客运量和货运量分别增长39.7%和191.2%。房地产开发完成投资增长22%，商品房销售面积225万平方米。赣州娱乐城基本建成。商贸零售、住宿餐饮等服务业均有较快增长。产业承接平台建设取得突破，开放水平明显提升。开工建设赣州香港产业园，规划面积50平方公里，首期开发11.7平方公里，一批香港及珠三角企业陆续落户园区，赣港合作进入新阶段。推出30条承接产业转移优惠政策，组建近千支招商小分队，开展承接产业转移百日对接活动。实际利用外资8.3亿美元，增长10.95%；实际引进内资209.29亿元，增长12.49%。光宝力信、王氏港建、曼妮芬、深圳亚太通等一批大项目落户赣州，亿元以上内资项目25个，新批500万美元以上外资项目59个，其中1000万美元以上项目15个。进出口总额12.6亿美元，增长37.6%，其中出口总额9.6亿美元，增长43%。赣州至深圳、厦门“铁海联运”集装箱班列运量大幅增长。

　　各项社会事业协调发展，可持续发展能力进一步提高。争取国家、省级科技项目28项和80项，申请专利346件，授权178件。数字城市建设进展良好。义务教育普及程度稳步提高，小学适龄儿童和初中适龄少年入学率达99.80%和95.55%，高中毛入学率提高4.8%。学校安全台账管理经验在全国推广。赣州中学、赣州师范高等专科学校开工建设。制作全国首部新农村建设题材电影《赣南之恋》。瑞金中央革命根据地历史博物馆开馆。市级广播电视公共覆盖工程建设加快。举办首届中国（赣州）地方戏展演、第二届全国“小康电视节目工程”颁奖等重大文化活动，赣南采茶戏首次走出国门赴新加坡交流演出。公共卫生、农村卫生、社区卫生能力增强，卫生监督基层网络建设稳步推进。深入开展“与奥运同行”为主题的全民健身活动，奥运火炬在瑞金传递活动圆满成功，竞技体育取得较好成绩。人口出生率控制在13.96‰以内，计划生育率达到80%。完成全市第二次农业普查。国防动员与后备力量建设得到加强。中心城区被评为“全国双拥模范城”，新一轮双拥创建深入开展。民族宗教、新闻出版、外事侨务、对台、气象、水文、监察、审计、统计、粮食、供销、档案、防震减灾、人防、地方志、慈善、红十字会、老龄、妇女儿童等各项工作取得新成绩。

　　“八项体系建设”快速推进。调研和论证工作深入开展，实施方案全部出台，城市应急物资储备体系建设正式启动，峰山应急水源工程和中心城区东出入口道路改造工程竣工。出台建立钨、稀土、萤石等优势资源储备体系相关文件，稀土储备工作有序推进。

　　环境保护和生态赣州建设进展良好。启动重点耗能企业能源审计、节能规划和节能电子档案创建工作。整治违法排污企业、保障群众健康等环保专项整治行动取得较好成效，关闭一批高能耗、高污染企业。主要河流断面水质、城市空气质量等环境指标保持良好水平，建立东江源国家级生态功能保护区试点和章江源、贡江源省级生态功能保护区。造林绿化“一大四小”工程建设全面展开。土地、矿产、森林等资源保护得到加强。

　　民生工程全面落实。农民人均纯收入增长9.1%，城镇居民人均可支配收入增长12.2%。省下达的81项民生工程指标全面完成。全年投入民生资金超过50亿元，增长90.1%。国家统筹城乡就业试点工作纵深推进，新增城镇就业12.85万人，新增转移农村劳动力19.27万人，“零就业家庭”动态安置率达到100%。在全国率先建设廉租店铺。城乡低保实现“应保尽保”，灾民救助及时有效，城乡困难群众临时生活救助制度全面建立，医疗救助51.2万人次。医疗保障“三张网”编制完成。新建、改建校舍50万平方米，农村学校D级危房全面消除。新建、续建廉租房22.19万平方米，中心城区和所有县城人均住房面积10平方米以下低收入家庭住房困难问题全部得到解决。新增经济适用住房23万平方米，在全省率先开工建设新市民公寓，完成19万平方米危旧房改造，农村困难群众住房救助受益农户3972户。解决21.6万人不安全饮水问题。减少贫困人口2.3万人，移民搬迁1.87万人。积极支持汶川等地震灾区抗震救灾和灾后重建，捐款3847.35万元，交纳“特殊党费”6393万元，捐物折价1991.6万元。

　　和谐稳定局面不断巩固。“五五”普法教育深入开展。县（市、区）委书记大接访及机关干部下访活动收效明显，信访渠道进一步畅通，社会矛盾纠纷化解力度加大。文明城市、文明村镇、文明单位、文明社区、文明风景旅游区（点）创建活动广泛开展。城乡社区建设扎实推进，章贡区中山路社区被评为“全国文化先进社区”。安全生产形势总体稳定, 实现事故起数和死亡人数双下降。和谐平安建设、基层综治工作、公众安全感满意指数居全省前列，赣州被评为“全国见义勇为先进城市”。

　　各位代表！过去的一年，是全市上下众志成城战胜年初历史罕见雨雪冰冻灾害，经济社会发展取得可喜成绩的一年；是我们有效应对复杂多变的宏观经济形势，为赣州改革开放三十周年写下浓墨重彩新篇章的一年；是全市各级政府按照市委的决策部署，在科学发展上攻坚克难、争先创优的一年；是全市人民在追赶与跨越的征途上扎实工作、奋勇拼搏、艰苦创业的一年。在这里，我代表市人民政府，向辛勤工作在各行各业的全市人民，向给予政府工作大力支持的各级人大代表、政协委员、各民主党派、工商联、无党派人士、人民团体，向驻市人民解放军、武警官兵、公安民警和离退休老同志，向所有关心、参与、支持赣州发展的建设者、劳动者和海内外朋友们，表示崇高敬意和衷心感谢！

　　在肯定成绩的同时，我们也清醒地看到发展中存在的困难和问题。主要表现在：经济总量较小，产业结构仍然不合理；全球金融危机对钨、稀土、氟盐化工、脐橙等四大产业集群和房地产业影响较大，部分产品价格下滑、销售形势严峻，农民增收、企业增效、财政增长的难度增大；工业化和城镇化水平不高，现代服务业发展相对滞后；土地、资源、环境容量约束加剧，转变经济发展方式的任务艰巨；部分群众生活较为困难，就业和社会保障工作仍需加强；政府职能转变、工作效率提高与新形势、新任务不相适应。对于这些问题，我们将高度重视，采取有力措施切实加以解决。

　　二、2009年经济社会发展目标任务

　　2009年，是新中国成立60周年、赣州撤地设市10周年，也是推进“十一五”规划顺利实施的关键一年。当前，历史罕见的国际金融危机对实体经济影响进一步加深，外需明显放缓，出口更加困难，外商投资增速下滑，企业经营困难增多，经济下行压力加大，但我国经济发展的基本面和长期趋势没有改变，发展的重要战略机遇期仍然存在。赣州经过改革开放30年特别是近年来一系列打基础、利长远的举措，积蓄了发展的能量和后劲，进入了又好又快发展的轨道。我们将认清形势，未雨绸缪，沉着应对，勇往直前，接受新考验，迎接新挑战，奋力开创赣州改革发展新局面，不负时代的重托和人民的期望！

　　今年政府工作的总体要求是：全面贯彻党的十七大、十七届三中全会和中央经济工作会议、省委十二届八次全会、市委三届六次全体（扩大）会议精神，以邓小平理论和“三个代表”重要思想为指导，深入贯彻落实科学发展观，纵深推进“一条主线，六个重点”工作部署，抓发展保企业，抓项目增投入，抓就业保民生，抓基础蓄后劲，保持经济平稳较快发展，全面推进社会主义经济、政治、文化、社会建设以及生态文明建设，把经济社会科学和谐的追赶与跨越推向新阶段。

　　2009年的主要预期目标是：生产总值增长12%；财政总收入增长13%，力争20%，地方一般预算收入增长11%；实际利用内、外资分别增长10%和5%，全社会固定资产投资增长30%，力争40%；城镇居民人均可支配收入增长9%，农民人均纯收入增长8.4%；城镇登记失业率控制在4%以内；人口自然增长率控制在8.03‰以内；单位生产总值能耗和工业增加值能耗分别降低4.2%和2%，化学需氧量排放有所降低；社会消费品零售总额增长15%，居民消费价格总水平涨幅控制在4%以内。

　　完成好全年目标任务，我们将重点把握以下几个方面：

——紧紧围绕发展这个硬道理，更加坚定攻坚克难的信心，以抓发展应对大挑战，以抓项目应对大困难，不为任何风险所惧，不被任何干扰所惑，凝聚积极作为的力量，形成共克时艰的气势，在困难中开辟发展的新天地；

——紧紧围绕保持经济平稳较快增长，更加清醒认识金融危机和国家扩大内需政策带给赣州的诸多发展机遇，努力从国际国内形势变化中发现和捕捉新的有利因素，善于在逆境中培育和运用赣州与沿海地区相比日益凸显的市场、资源、劳动力、成本等方面的优势。化危机为生机，变压力为动力，变经济波动期为发展机遇期，把握发展的主动权，迎接发展的新春天；

——紧紧围绕增强发展的动力和活力，更加积极深化改革开放，创新体制机制，创优发展环境，广泛集聚各类发展要素，特别是扶持有竞争力的企业低成本扩张和参与重组洗牌，加速发展壮大；

——紧紧围绕做大产业、做强基础，更加主动调整结构和转变发展方式，加大有效投入，推动产业优化升级，加强基础设施建设，统筹推进新型工业化、新型城镇化和社会主义新农村建设；

——紧紧围绕富民惠民安民，更加注重保障和改善民生，把群众的安危冷暖放在心上，着力扶持就业创业，切实解决涉及群众利益的难点热点问题，努力使全市人民过上幸福安康的生活。

　　（一）推进项目建设，在有效投入上有更大突破

　　抓住国家扩大内需政策机遇上项目。加大重大项目前期工作力度，加强对交通、城市基础设施、产业化等项目的调度，帮助解决项目建设中的资金、土地、施工环境、项目报建等问题。完善项目建设推进机制，着力加快项目建设进度，加强对投资5000万元以上重大项目的管理。进一步明确重点工程目标责任，落实领导责任制、过错追究制和奖惩激励机制，坚持一个项目、一个领导、一套人马、一抓到底，坚决把项目抓成抓好。

　　推进以交通为重点的基础设施建设。全年市属重点工程项目106个，总投资920.9亿元，年度计划投资242.8亿元。开工建设厦蓉高速隘瑞段和赣崇段、济广高速瑞寻段、大广高速龙南里仁至赣粤省界段，总里程691公里。推动赣韶铁路尽快开工，组织实施赣州火车站客运站改扩建工程，新建赣州东站集装箱二类口岸站。争取早日开工赣龙铁路复线改造、鹰瑞汕铁路、赣州至井冈山铁路等项目。加强边远山区县交通改造升级，建设农村公路3000公里，国、省道公路新建及改扩建209公里。

　　 （二）推进十大体系建设，在体制机制创新上有更大成效

　　密切关注国家宏观调控政策和沿海发达地区政策走势，不断丰富完善十大体系配套文件。加快出入境检验检测中心、电子口岸等物质载体项目建设。继续做好十大体系建设“三进”工作，加速体制机制创新成果转化。完成赣州钴钨公司等企业破产清算工作，引资重组赣南制药厂、赣州高速公路公司等企业，推进国有企业产权、用人和分配制度改革。深化预算、非税收入收缴、政府采购和收入分配制度改革。稳步推开事业单位岗位设置管理工作。加快国家“教育综合改革实验区”建设。推进农村教育、医疗卫生等公共服务体系和养老保险等社会保障体系改革，探索适合赣州实际的土地承包经营权流转形式。加快林权配套改革，将市县林业产权交易中心纳入招投标中心建设。

　　（三）推进产业集群建设，在工业扩量提质上有更大作为

　　注重发展内涵，注重技术创新，注重结构调整，注重产业升级，力促工业经济迈上新台阶。全年规模以上工业增加值、主营业务收入分别增长20%以上，规模以上工业主营业务收入力争突破1000亿元，工业增加值占GDP比重提高2个百分点。

　　采取强有力措施扶持现有企业稳定发展。认真落实中央、省、市有关减轻税费、节能减排等各项扶持政策，推动企业加强产品、技术和经营创新，提高企业生存能力和抗风险能力。制定实施企业减负相关措施，切实减轻企业负担，帮助企业渡过难关。严格控制钨、稀土、萤石开采总量，适时运用收储、限产保价等措施，稳定市场，增强企业发展信心。

　　继续大力推进“五个十百亿”工程。建立“五个十百亿”工程责任制和目标考核制。加大资源整合力度，打造大余、崇义、上犹和赣州东片等钨资源板块，加快稀土矿业公司转型及与战略投资者联合重组，推进氟化工产业龙头对萤石矿山兼并联合，发展精深加工及应用产品。壮大钨、稀土、氟盐化工三大优势产业集群，抓好有色产业深加工基地、氟盐化工产业基地建设。加大扶持力度，培育形成电子电器、铜加工、新能源新材料及农副产品加工等新兴产业集群。制定实施三大优势产业和六大主导产业招商规划，策划一批符合国家产业政策的重大项目，启动建设一批有利于产业升级的重大项目。集中力量推进重点产业项目落户和开工建设，对投资10亿元以上项目实行市领导挂点一对一跟进服务。抓好全民创业和中小企业成长工程，力争年内净增规模工业企业100户以上。

　　加强工业协调服务。强化工业经济运行监测和调度，建立企业服务联动机制，大力扶持100户重点企业。及时跟踪要素市场供求变化，抓好煤电油运和重要生产资料保障。完善生产性服务体系，加强公共技术服务平台软硬件建设，完善国家钨与稀土产品质量监督检测中心建设，支持帮助企业提高创新能力，创立自主品牌，拓展国内市场，为企业低成本、高效益发展营造良好环境。

　　（四）推进社会主义新农村建设，在县域经济发展上有更大力度

　　整体推进“五新一好”。全面启动县域镇村布局规划编制，通盘谋划城乡一体化建设发展。继续抓好“五新一好”整体推进重点乡镇工作。完成2000个新增建设点的村庄整治和60个新增试点乡镇的圩镇整治建设任务，抓好1000个完善提升点。积极开展新型村落社区建设，不断丰富村民自治的有效实现形式。加快农民教育培训网络体系和乡镇农民综合服务中心建设。

　　大力发展现代农业。扶持农夫山泉、斌杰食品、谱赛科生物技术、华劲纸业等一批龙头企业做大做强。启动新一轮优势农产品区域布局规划，发展加工甜橙、生猪、油茶、烟叶、甜叶菊、造纸工业原料林等优势主导产业，大力引进农产品深加工企业。加强农民专业合作经济组织规范化建设，充分发挥供销社系统在农产品营销中的作用，完善脐橙营销体系建设。推进农产品品牌建设，积极做好“绿色食品”“有机食品”申报工作。加强农业基础设施建设，推进病险水库除险加固、灌区配套与改造工程。

　　加快发展县域经济。科学利用资源兴工、突出产业招商兴工，以工业化思维发展各具特色的县域经济。瞄准国际市场和沿海发达地区，因地制宜发展资源经济、配套经济、劳务经济、生态经济，为沿海工业和服务业提供产业配套及产品供给，优化特色农业产业区域布局，形成“一村一品、一县一业”发展模式。

　　（五）推进现代服务业发展，在拉动消费需求上有更大举措

　　加快金融业改革发展，千方百计扩大信贷规模，加大对优势产业、特大城市构建、基础设施建设等方面的信贷支持力度。整合重组担保公司，破解企业、城乡居民抵押难和担保难等问题。加快企业上市进程，组建赣州发展投资集团，做好发行企业债券工作，争取设立首支创业投资基金。消除金融服务盲点，加大股份制商业银行引进力度，做好交通银行落户筹备工作，加快赣州银行机构延伸和跨区域经营步伐。抓好市旅游集散中心和马祖岩旅游景区建设，办好第三届赣州生态旅游文化节等旅游活动，力争在景区创A、饭店创星上取得突破。推进以综合物流园区和六大节点为核心的赣州城区物流体系建设，培育和引进一批上规模、有实力的物流企业。促进房地产业平稳健康发展，扩大住房投资和消费，建设赣州房地产交易中心，搞活房地产二级市场。加快繁荣信息、商贸、文化娱乐等服务业。

　　（六）推进招商引资和产业承接平台建设，在开放型经济水平上有更大提高

　　全年实际利用外资达到8.7亿美元，引进内资237亿元，引进5000万元以上工业项目120个；外贸进出口总额达到15亿美元，其中出口达到11.55亿美元。一是围绕产业招商。突出抓好钨、稀土、氟盐化工等产业集群和六大主导产业招商，加大旅游、现代农业招商力度。对每个重点招商产业，采取一名市领导挂点、一个牵头部门负责、一笔招商经费保障、一支小分队参与、若干个相关部门配合、一至二个主承接地对接的招商方式，增强招商引资的针对性和实效性。二是创新思路招商。传统招商与专业招商并举，乡情招商、协会招商、商会招商并进，加强入港、入台招商，主攻重点区域招商。稀土精深加工以日本、韩国为重点，钨精深加工以美国、欧洲为重点，大型国有企业以北京为重点，国内大型民企以长三角、珠三角为重点。三是推动园区招商。大规模拉开香港产业园建设框架，加快出口加工区标准化厂房及配套生活设施建设。引导各工业园区结合自身实际，根据资源禀赋和产业基础择商引资，建设各具特色的园区，形成以香港产业园为“龙头”，适合各类投资项目需求的园区发展格局。四是立足企业招商。依托现有钨、稀土、氟盐化工民营企业，大力引进合作伙伴、上下游配套项目以及相关产业，支持企业增资扩股扩产，扩大经营规模。

　　（七）推进特大城市构建，在新型城镇化上有更大进展

　　围绕中心城区3年至5年内建设成为100万人口特大城市目标，分片区、分阶段推进特大城市构建，实现城市面貌一年一变样，三年大变样。一是完善特大城市规划。修编《赣州市城市总体规划》，迅速拉开特大城市框架。加快都市区内产业布局、重要项目、重大基础设施、土地利用等规划对接。分功能片区完善章江新区、河套老城区、开发区等六大片区规划，构建资源共享、优势互补、功能齐全、协调发展的组团式生态型都市新区。对城市整体形象及建筑物进行精品设计，打造地方特色鲜明、富有文化品位和彰显艺术个性的城市雕塑景观和城市夜色景观。二是推进特大城市建设。全面启动六大片区项目建设，开工建设贡江大桥、新世纪大桥，完成飞龙岛大桥、章江大桥、红都大道、章江南岸防洪堤一期工程等路桥建设，基本形成章江新区路网框架。加快赣南大道建设，启动赣州监狱搬迁工程。建成中央生态公园和杨梅渡公园二期。加强赣县城区、南康城区、章贡区及赣州经济技术开发区的公交对接，加速赣州都市区公交一体化进程。三是提高城镇化水平。积极探索人口向城镇集中、务工人员向工业园区集中、土地向种养能手和大户集中、就学向县城和乡镇集中等“四个集中”的有效途径，促进人才、劳务、技术、资金等生产要素向城镇集聚。推进瑞金、龙南次中心城市建设。搞好城镇规划，拉开城镇框架，加大县城和中心镇建设力度，全市城镇化率达40%以上。四是加强城市经营管理。扎实开展城市管理年活动，加大中心城区公共交通秩序整治力度。建立属地化城市管理模式，市级调度建设，区级管理服务。推动城管行政执法相对集中，健全综合执法与专业执法、统一执法与分级执法、日常执法与专项整治相结合的执法体制。创新投融资体制，鼓励各类投资主体参与市政公用设施建设运营。推进文明城市、卫生城市创建工作。南康创建国家生态园林城市，3个以上县（市）创建省级生态园林城市。

　　（八）推进以改善民生为重点的社会建设，在促进全面协调可持续发展上有更大步伐

　　深入实施民生工程，着力为民办实事。坚持新增财力向困难群众、农村、基层、公共社会事业倾斜，全面完成省政府确定的“八个重点”、81项指标任务，着重办好十个方面实事。一是高度重视返乡农民工问题，做好返乡农民工培训工作，积极扶持返乡农民工创业就业，全年培训农民50万人次。城镇新增就业人口5.4万人，新增转移农村富余劳动力9.1万人。组建小额贷款公司，发放小额贷款2.23亿元。新建设廉租店铺400个。二是开展失业保险市级统筹试点和新型农村社会养老保险试点工作，抓紧解决部分群体未参加养老医疗保险问题，扩大被征地农民参加养老保险试点范围。三是城乡低保实现“应保尽保”“应退尽退”，月人均补差水平城市低保达到150元，农村低保达到60元。加大“三院”改扩建力度，提高农村五保集中供养率，健全临时生活救助制度，做好城乡困难群众医疗救助工作。四是为121万名城乡义务教育阶段中小学生免除学杂费和免费提供教科书，为6万名以上义务教育阶段贫困寄宿生补助生活费。五是实现医疗保障全覆盖，城镇职工基本医疗保险、工伤保险参保人数分别达到53.9万人和24.56万人,城镇居民基本医疗保险覆盖率达到100%，新型农村合作医疗参合率达到90%以上。六是所有县（市）建立农产品检测分中心，30%乡镇建立农产品检测室。加大食品药品全过程监管力度，提高安全控制度。七是新建廉租房25万平方米，其中中心城区10万平方米；中心城区新建、续建经济适用房14万平方米，建成新市民公寓1000套；改造棚户区30万平方米；实施好农村困难群众危旧房改造工程。启动30座渡改桥项目建设。八是加快第二污水处理厂和章江新区污水管网及干管、第二垃圾处理场建设步伐，确保中心城区第一污水处理厂和所有县（市）污水处理设施正式运行。九是抓好1047个重点村整村推进扶贫开发，完成1.87万人移民扶贫搬迁，农村绝对贫困人口递减10%以上。十是完善乡镇文化站建设，全市每个乡镇有一个影剧院或演出场所；加强农村社区体育健身场所建设；抓好741个“村村通”工程建设，扩大市本级广播电视节目覆盖率。

　　加快“八项体系建设”，建立健全科学发展长效机制。按照实施方案要求，强化落实责任，加强协调调度，迅速启动一批“八项体系建设”项目，全力推进城市交通应急通道、城市应急饮用水源等体系已开工项目建设，加快各体系实体工程建设进度，力争年内取得更大成效。

　　加强环境保护，打造绿色生态赣州。强化落后产能淘汰机制和节能减排措施，建立节能减排目标责任制，深入开展污染整治工作，加强环境执法监管，严格执行环境影响评价、建设项目“三同时”和环境准入制度，落实主要污染物排放总量控制和企业排污许可制度。加大对重点流域区域的环境监管力度，对已无环境容量的流域区域实行区域限批。建立县（市、区）主要河流过境断面水质监测考评机制，实行水资源质量辖区负责制。发展循环经济，推进绿色生态创建工作，切实做到绿色生态“五个零排放”。控制农村面源污染。以“一大四小”工程建设为重点，加大造林绿化力度，加强林地保护管理。开展生态自我修复，加快水土流失治理步伐。加强耕地资源保护，严格用地管理，提高土地集约节约利用水平。

　　加快文化、教育、卫生、体育等各项社会事业发展，促进社会和谐稳定。着力打造宋城文化、客家文化、红色文化品牌，提升文化服务经济社会发展的软实力。做好文化艺术中心建设前期筹备工作，加强文化遗产保护工程建设。推进“科教兴市”战略，办人民满意的优质教育。抓好义务教育合格学校建设和学校卫生“达标创星”工作，加强农村寄宿制学校建设。创建市属高等职业技术学院，加快赣州中学、赣州师范高等专科学校建设，加大赣州技师学院、赣州人民医院迁建工程建设力度。加强公共卫生、农村卫生和社区卫生体系建设，提高医疗服务质量。广泛开展全民健身运动，推进农民体育健身工程，抓好峰山休闲体育公园建设，推动竞技体育上新水平。围绕稳定低生育水平、统筹解决人口问题，进一步抓好新时期人口计生“五项重点工作”。认真组织实施第二次全国经济普查工作。全面落实安全生产责任制，突出抓好生产、交通、防火三大领域的安全工作，确保实现“两杜绝、一减少”目标。强化综治维稳工作，畅通和拓宽信访诉求渠道，积极预防和妥善处理群体性事件，深入推进和谐平安赣州建设。

　　三、加强政府自身建设

　　过去的一年，面对年初历史罕见雨雪冰冻灾害，全市各级政府和广大人民群众快速反应、紧急动员、周密部署、有力处置，最大限度地降低了灾害损失，夺取了灾后重建全面胜利。及时出台扩大投资刺激消费的一系列政策措施，积极应对全球金融危机对我市的影响。完善政府工作制度，科学民主决策水平和行政透明度不断提高。行政审批制度改革进一步深化，全市行政许可事项减少近200项，窗口办事效率明显提高，市县行政服务中心行政审批服务办结率及群众满意率均达98%以上。办复人大代表建议179件，政协委员提案349件。认真落实《政府信息公开条例》，市县政府主动公开政府信息6.12万条。对市级政府24个重点建设项目进行了专项监督检查。开展“我与市长话和谐”主题热线节目、政风行风热线活动和民主评议政风行风、市长热线工作，反响较好。我市行政复议工作获全国先进。加强机关作风效能建设，经济发展环境进一步优化。从源头上防止腐败的力度加大，勤政廉政建设得到加强。

　　做好2009年各项工作，政府肩负着重要责任。我们一定以强烈的事业心和责任感，推进赣州的改革开放和现代化建设事业。

　　提升行政效能。在全市政府系统深入开展“机关效能年”活动，推进并联审批、“两个集中”试点，简化项目审批程序，缩短审批时限，做到急事急办、特事特办。切实落实行政问责制，大力整治行政不作为、办事拖拉、效率低下等问题，加强督促检查和行政效能监察，确保各项政策措施落到实处。切实精简会议，压减文件数量，减少迎来送往，推进政府工作大提速。

　　提升破难能力。着力把握发展新形势和全市工作大局，增强忧患意识，努力做到善“预难”，对困难及早预料，有充分估计和准备；不“避难”，对各种矛盾和问题不绕道、不退缩、不畏惧，坚信办法总比困难多；敢“破难”，勇于迎着困难上，积极用改革创新的思路和办法破解增长难、融资难、用地难、拆迁难等问题。

　　提升管理水平。密切关注经济动态，适时调控经济运行，维护市场经济秩序。健全科学民主决策机制，完善公众参与、专家论证和政府决策相结合的决策体系，强化重大事项集体决策制度。推进依法行政，做好行政复议工作。加强电子政务建设，推进政府信息公开，提高行政透明度。完善行政服务中心建设和管理。自觉接受市人大及其常委会的法律监督和工作监督，接受市政协的民主监督，做好人大代表建议、政协委员提案办理工作。广泛听取民主党派、工商联、无党派人士和各人民团体的意见和建议。运用审计结果，加强审计监督。

　　提升群众满意度。加强公务员队伍建设，增强各级政府的责任意识、服务意识和节俭意识，勤政务实、廉洁从政。建设 “12345”政府热线，办好市长热线、市长信箱，坚持问政、问计、问需于民。加强调查研究，真诚倾听群众呼声，真实反映群众愿望，真情关心群众疾苦，善办好事、多办实事，努力建设人民满意的服务型政府。

　　各位代表，赣州正处于追赶与跨越的重要历史时期，我们深知责任重大，使命光荣。让我们高举中国特色社会主义伟大旗帜，更加紧密地团结在以胡锦涛同志为总书记的党中央周围，以科学发展观统领全局，在中共赣州市委的坚强领导下，同心同德，奋发有为，为开创赣州更加美好的未来而努力奋斗！
